# The synthesis of copper-modified biochar from *Elsholtzia Harchowensis* and its electrochemical activity towards the reduction of carbon dioxide

**DOI:** 10.3389/fchem.2023.1238424

**Published:** 2023-08-30

**Authors:** Shiqi Chen, Wei Liu, Ziwei Mei, Haifu Li, Wenyu Zhao, Junkai Zhao, Hong Tao

**Affiliations:** Department of Environmental Science and Engineering, University of Shanghai for Science and Technology, Shanghai, China

**Keywords:** *Elsholtzia Harchowensis*, biochar, electrocatalysis, CO_2_RR, carbon-based materials

## Abstract

Phytoremediation techniques have been widely used in the treatment of heavy metal contaminated soils in recent years, but there is no effective post-treatment method for plant tissues containing heavy metals after remediation. *Elsholtzia Harchowensis* is a copper hyperaccumulator, commonly distributed in copper mining areas and often used for soil remediation of mine tailings. Moreover, copper-based catalysts are widely used in electrocatalytic reduction of carbon dioxide, which aims to convert carbon dioxide into useful fuels or chemicals. In this study, copper-modified biochar was prepared from *Elsholtzia Harchowensis*. Its specific surface area can reach as high as 1202.9 m^2^/g, with a certain porous structure and even distribution of copper on the amorphous carbon. Various products (such as carbon monoxide, methane, ethanol, and formic acid) could be obtained from the electrolytic reduction of carbon dioxide by using the as-prepared catalyst. Instantaneous current density of up to 15.3 mA/cm^2^ were achieved in 1.0 M KHCO_3_ solution at a potential of −0.82 V (vs. RHE). Electrolysis at a potential of −0.32 V (vs. RHE) for 8 h resulted in a stable current of about 0.25 mA/cm^2^, and the Faraday efficiency (FE) of carbon monoxide can reach as high as 74.6%. In addition, electrolysis at a potential of −0.52 V (vs. RHE) for 8 h led to a stable current of about 2.2 mA/cm^2^ and a FE of 8.7% for the C_2_ product. The rich variety of elements in plants leads to catalysts with complex structural and elemental characteristics as well, which facilitates the electrolytic reduction of carbon dioxide with a variety of useful products.

## 1 Introduction

In recent years, heavy metal pollution in soil has attracted more and more attention, mainly including copper (Cu), zinc (Zn), chromium (Cr), cadmium (Cd), lead (Pb), mercury (Hg), arsenic (As) and nickel (Ni). The soil was contaminated with industrial and agricultural activities, resulting in much higher heavy metal content than the natural background value and leading to ecological degradation and soil quality deterioration (Adrees et al., 2015; Sarwar et al., 2017). China has very rich reserves of mineral resources, but due to improper mining methods and other problems, the problem of heavy metal pollution of soil caused by mining is becoming increasingly prominent. Phytoremediation is an emerging environment-friendly remediation technology, which has a wide remediation area, low cost, small disturbance to soil, convenient operation and simple management, and no secondary pollution. Furthermore, it increases the vegetation cover of the mining area, which can effectively protect the surface soil and reduce soil erosion, and has important practical significance and strong operability for the ecological restoration of the soil around the mining area and the abandoned land of the mining area as well as the remediation of the heavy metal contaminated soil in the mining area (Alvarenga et al., 2009; Grandlic et al., 2009; Barceló and Poschenrieder, 2011; Rascio and Navari-Izzo, 2011).

*Elsholtzia Harchowensis*, commonly known as copper grass flower, is an annual herb in the family *Labiatae*, which can grow in soils with high copper content and is regarded as an indicator plant for copper mines. The plant has been applied in the field of environmental pollution management, mine site revegetation, plant mineral search and tolerance mechanism to heavy metals (Song et al., 2003; Jian et al., 2004; Jiang et al., 2004). Nowadays, *Elsholtzia Harchowensis* is one of the main remediation plants for copper-contaminated soils in China, and post-treatment of plant tissues containing heavy metals is a key problem to be solved, as it is very likely to cause secondary contamination if not handled properly (Li et al., 2020). In Tongling City, the total area of the mine is estimated to be around 2,688 hectares, and the phytoremediation treatment area is up to 1000 ha. The area has adopted *Elsholtzia Harchowensis* to remediate the soil contaminated by the mine, with 5.3 ha of *Elsholtzia Harchowensis* planted around the tailings pond alone, producing a large number of plants containing the heavy metal copper. Due to the low copper content, the economic benefits of incineration to recover copper are not obvious. The current treatment methods still focus on combustion and composting, which can cause secondary pollution to the air and soil. Therefore, how to effectively treat these plant tissues containing heavy metal copper has become a great challenge.

On the other hand, with the rapid development of industry, CO_2_ emissions have increased significantly, seriously disrupting the carbon cycle and even the balance of the entire ecosystem in the Earth’s environment (Wang et al., 2011). As a greenhouse gas, the utilization of CO_2_ contributes to sustainable economic and social development, and its conversion into fuels or chemicals with high added value can not only alleviate the energy crisis (Sun et al., 2021; Niu et al., 2022), but also achieve a closed carbon cycle and mitigate the greenhouse effect (Mac Dowell et al., 2017; Birdja et al., 2019; Tang et al., 2021). Various strategies (such as electrocatalytic reduction (Costentin et al., 2013; Qiao et al., 2014; Nitopi et al., 2019; Ross et al., 2019), thermocatalytic hydrogenation (Markewitz et al., 2012), photoelectrocatalysis (Fu et al., 2012; Habisreutinger et al., 2013) etc.) are investigated in the past decades for the conversion of CO_2_ to useful products. Among those methods, electrocatalytic reduction of CO_2_ enables both renewable electricity storage and direct conversion of CO_2_ into value-added products such as CH_4_, C_2_H_4_, C_2_H_5_OH, and HCOOH (Hori, 2008; Franco et al., 2020; Zhang et al., 2023).

In the electrocatalytic CO_2_RR studied so far, the non-precious metal Cu has attracted much attention because of its unique catalytic properties (Chen et al., 2021; Chen et al., 2022b). Cu materials are cheap and exhibit unique catalytic properties in electrocatalytic CO_2_ reduction reactions, producing a wide range of hydrocarbons and alcohols with high current efficiencies. For instance, CO_2_ could be electrochemically reduced to CH_4_ and C_2_H_4_ on the surface of metallic Cu as reported by Hori et al. (Hori et al., 1986; Zhu et al., 2013). Among all the reduction products of CO_2_RR, multi-carbon (C_2_^+^) products, such as ethylene and ethanol, have a higher energy density as well as additional commercial utilization value. In addition, high overpotential is needed to achieve large current density as well as fast reaction rate for CO_2_ conversion, which enhances the hydrogen evolution reaction (HER) at the same time. To solve this problem as well as to improve the selectivity of specific products, novel electrodes have been developed recently such as bimetallic materials (Au-Cu (Zheng et al., 2018), Ag-Cu (Fu et al., 2022), Pd-Cu (Costentin et al., 2014)), molecular sieve catalysts (Liu et al., 2018), carbon based materials (Wang et al., 2017; Liu et al., 2022), and transition metal single-atom catalysts (SACs) (Wang et al., 2018; Sun et al., 2021; Chen et al., 2022a) etc.

Carbon based materials have attracted more and more attention world widely due to simple preparation method, low cost, high catalytic ability and stability (Wang et al., 2021). Recently, heteroatom doping has been considered an effective strategy for improving electrocatalytic performance (Asefa, 2016; Wang et al., 2019; Zhang et al., 2019; Liu et al., 2023). For carbon materials, S dopant has been proved to be able to adsorb the generated H* at reduction potential and promote the dissociation of water, so it has attracted extensive attention of many researchers (Ma et al., 2019; Sun et al., 2020; Zheng et al., 2021). Biochar material is a good carrier with electrical conductivity and can be modified by doping to obtain electrocatalysts with large specific surface area and highly dispersed active components, which have broad application prospects. In this study, *Elsholtzia Harchowensis* collected in Tongling, China, was used to prepare carbon-based catalytic materials from copper-containing biomass. Exploring the use of copper accumulated in plant cells for electrocatalytic conversion of carbon dioxide is expected to utilize waste *Elsholtzia Harchowensis* and obtain considerable benefits for the environment. The modified biochar was prepared by carbonization at high temperature and then activation by KOH. The differences in the electrocatalytic performances of biochar prepared by untreated *Elsholtzia Harchowensis* and CuSO_4_ soaked plant tissues were compared. Since the sulfur-doped Cu-based biochar was obtained by soaking the CuSO_4_ solution-treated material and introducing both Cu and S into it, the influence and correlation of non-metallic elements on its performance and structure for electrocatalytic reduction of CO_2_ were also investigated in this study.

## 2 Experimental

### 2.1 Experimental reagents and materials

Copper sulfate pentahydrate (CuSO_4_·5H_2_O, AR), potassium hydroxide (KOH, AR), anhydrous sodium sulfate (Na_2_SO_4_, AR), anhydrous ethanol (CH_3_CH_2_OH, AR), potassium bicarbonate (KHCO_3_, AR) were purchased from Sinopharm Chemical Reagent Co., Ltd. Nafion solution (5% D520) was purchased from Suzhou Yilongsheng Energy Technology Co., Ltd. All reagents were of analytical grade and were used as received without further purification. *Elsholtzia Harchowensis* was collected from the copper mine area of Tongling City, Anhui Province, China.

### 2.2 Preparation of catalysts

#### 2.2.1 Biochar (BC)

The collected *Elsholtzia Harchowensis* was cleaned, dried and crushed after sampling, and then a 40 mesh sieve was used to obtain powdered plant tissue. Carbonization was performed in a tube furnace under specific conditions of nitrogen atmosphere, with a heating rate of 2 °C/min to 700 °C, and maintained for 90 min. After cooling, the samples were ground and processed. Potassium hydroxide powder was added at a mass ratio of 1:4 (carbon: KOH) and mixed thoroughly, after which the mixture was placed again in a tube furnace at a heating rate of 2 °C/min to 700 °C under a nitrogen atmosphere for 90 min to obtain activated biochar material (Abbas et al., 2022). After cooling, the sample was washed with pure water until neutral, dried, and ground in an agate bowl to obtain pristine biochar (BC).

#### 2.2.2 Copper doped modified biochar (Cu/C-S)

**FIGURE 1:**
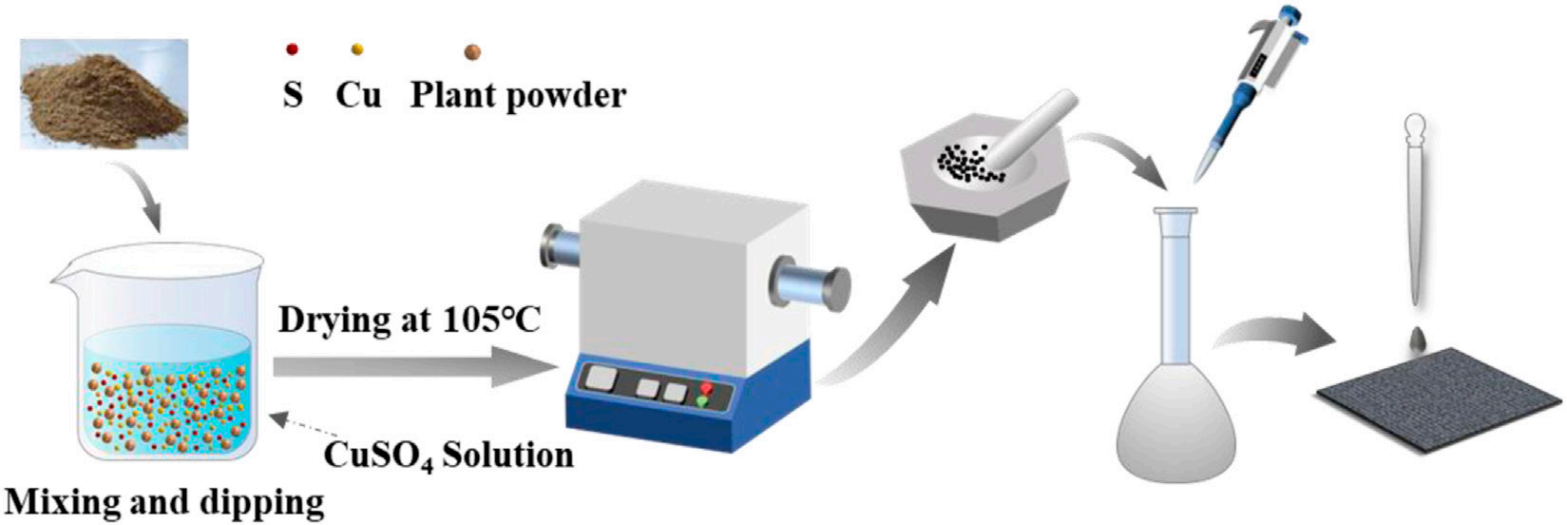
Schematic diagram of the preparation of Cu/C-S electrode material.

The powdered plant tissues after passing through a 40 mesh sieve were added to copper sulfate pentahydrate in a mass ratio of 1:3 (copper: *Elsholtzia Harchowensis*), dissolved in 30 ml of pure water and mixed thoroughly. After stirring with a magnetic stirrer for 2 hours, the beaker was sealed with a sealing film and kept for 24 h. After that, the slurry was dried in the oven at 105°C, and then charred at 700°C for 90 min at a heating rate of 2°C/min under the nitrogen atmosphere using the tube furnace. The product was mixed with potassium hydroxide powder at a mass ratio of 1:4 (charcoal: KOH) after cooling and mixed thoroughly, then activated at 700°C for 90 min. The sample was washed with pure water until neutral, then dried and ground in an agate bowl to obtain copper doped modified biochar (Cu/C-S) as shown in Figure 1.

### 2.3 Working electrodes

7 mg of catalyst and 100 μl of 5% Nafion solution were mixed in 1 ml of anhydrous ethanol and sonicated for 1 h to make the catalyst dispersed uniformly. Then the mixed slurry was coated on a 1 × 1.5 cm hydrophobic carbon paper in multiple drops with a catalyst loading of 2.4 mg/cm^2^, followed by drying at room temperature. Before testing, the working electrode was immersed in a 0.2 M Na_2_SO_4_ solution, and then reduced for 3600 s at a potential of −0.82 V (vs. RHE) for activation.

### 2.4 Catalyst characterization and testing

#### 2.4.1 Material characterization

The X-ray diffractometer (XRD, Malvern Panalytical Empyrean, Netherlands) was using to determine the crystal structure of the as-prepared catalyst using a Cu target as the radiation source, at a scanning speed of 2°/min from 10° to 80°. The microscopic surface morphology as well as element distribution was observed with a field emission scanning electron microscope (FE-SEM, ZEISS GeminiSEM 300, Germany) equipped with energy dispersive spectrometer (EDS, ZEISS GeminiSEM 300, Germany). For the test with transmission electron microscopy (TEM, JEOL JEM-F200, Japan), the catalysts were firstly dispersed in ethanol solution, and then added dropwisely to a copper grid, followed by photographing with an accelerating voltage of 200 kV. The chemical states of possible elements were analyzed with X-ray photoelectron spectroscopy (XPS, Thermo Scientific ESCALAB 250Xi, United States) using a monochromatized Al-Kα source (Mono Al-Kα) with hv = 1486.6 eV. The calibration was performed using C 1s with an binding energy of 284.8 eV. Fourier Transform infrared spectroscopy (FT-IR, Thermo Scientific Nicolet iS5, United States) samples were employed to determine the possible functional groups with a range of 400–4,000 cm^-1^. The automatic specific surface and porosity analyzer (BET, Micromeritics 3Flex, United States) was used to characterize the pore characteristics and surface area with a desorption temperature of 200°C and a degassing time of 8 h. The specific surface area was calculated by Brunauer Emmett Teller (BET) method, and the pore size characteristics were calculated by Barrett Joyner Halenda (BJH). Determination of metal elements in materials by inductively coupled plasma-mass spectrometry (ICP-MS).

#### 2.4.2 Electrochemical test

The electrocatalytic CO_2_RR was carried out in an H-type electrolytic cell with the cathode chamber separated from the anode chamber by a DuPont N-117 proton exchange membrane, and the volume of electrolyte in both chambers was 50 ml. Platinum sheets (2 × 2 cm) and Ag/AgCl electrodes were used as the counter and reference electrodes, respectively. All electrochemical tests were performed with automatic 95% iR compensation using an electrochemical workstation (CHI660E, Chenhua Instrument Co., Ltd., Shanghai, P. R. China). Before the test, high-purity argon gas (99.999%) was introduced into the cathode chamber for 30 min to exhaust the air from the system, and then high-purity carbon dioxide (99.99%) was introduced for 30 min to saturate the electrolyte with carbon dioxide. The experiment was conducted with a continuous gas flow of carbon dioxide at a rate of 20 ml/min.

In the cathode gas product, hydrogen gas was detected using gas chromatography (GC-9890B, Shanghai Linghua Instrument Co. Ltd., China) with a carrier gas of 99.9% high-purity argon gas, a gas quantitative sampling tube capacity of 1 mL, a thermal conductivity detector (TCD) temperature of 80°C, and a column box temperature of 120°C; Carbon monoxide, methane, and carbon dioxide were chromatographically analyzed (GC 2060, Shanghai Ruimin Instrument Co. Ltd., China) with 99.9% high-purity nitrogen as the carrier gas. The injector temperature was 150°C, the column temperature was 50°C, the hydrogen ion flame detector (FID) temperature was 150°C, and the conversion furnace temperature was 330 °C. The liquid products were detected by 1H nuclear magnetic resonance spectrometer (NMR, Bruker 400M, Germany). The electrolyte solution (1000 μL) was sampled for NMR analysis, and DMSO (40 μL) was used as internal standard.

## 3 Results and discussion

**FIGURE 2:**
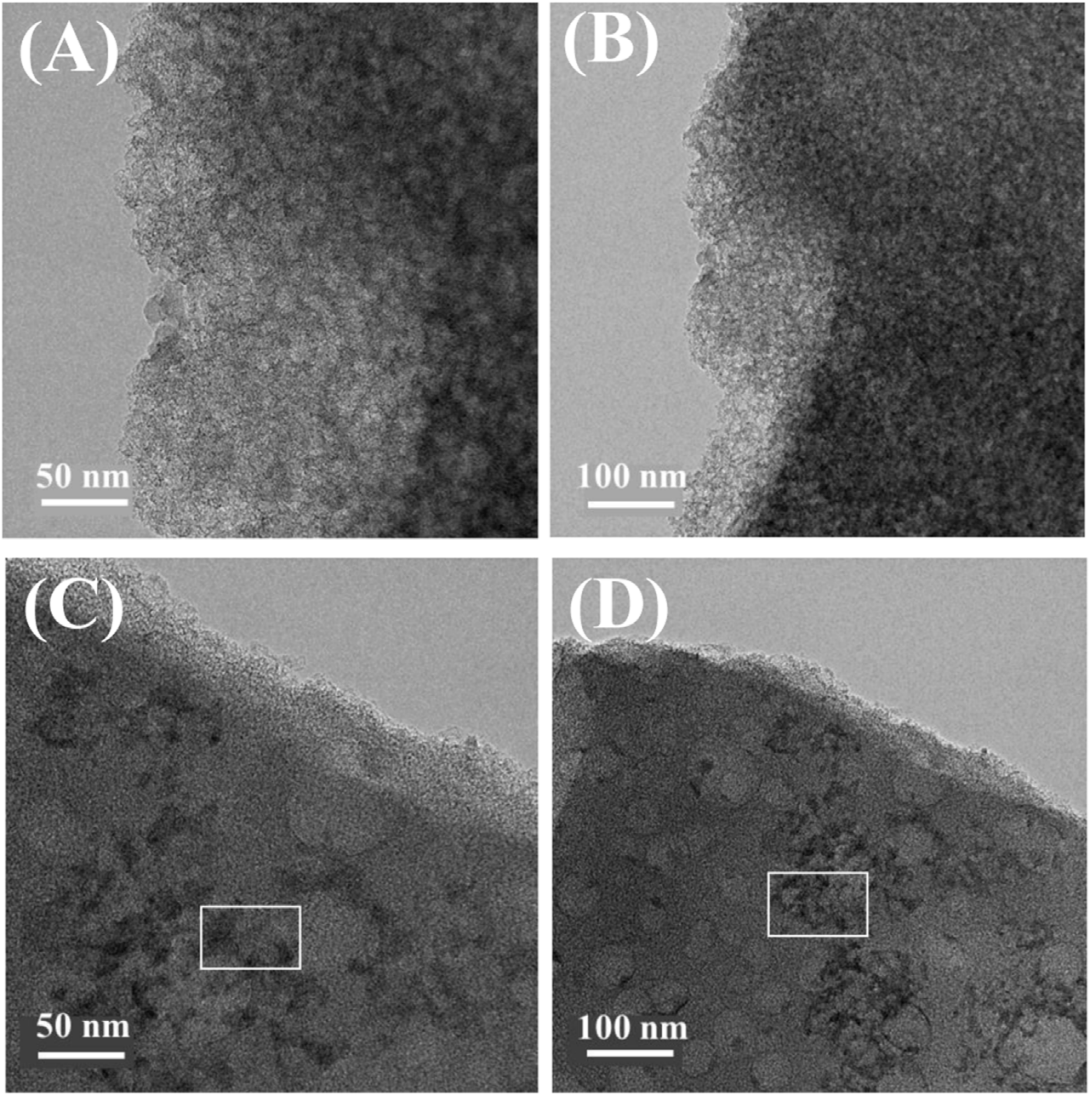
TEM images of BC and Cu/C-S materials: **(A)** and **(B)**: BC; **(C)** and **(D)**: Cu/C-S.

The morphological characteristics of the as-prepared materials were investigated using transmission electron microscopy (TEM), respectively, and the results were shown in Figure 2. Figures 2A, B are TEM images of pristine BC material, while C and D are TEM images of Cu/C-S material. Obviously, both materials were granular in shape with uniform surface distribution and had a good pore structure. Moreover, the particles of Cu/C-S material were darker in the center and brighter at the edges, which indicated that the structure of the catalyst was thicker in the center and decreased gradually at the edges, and it was possible that the black particles on top were copper compounds.

**FIGURE 3:**
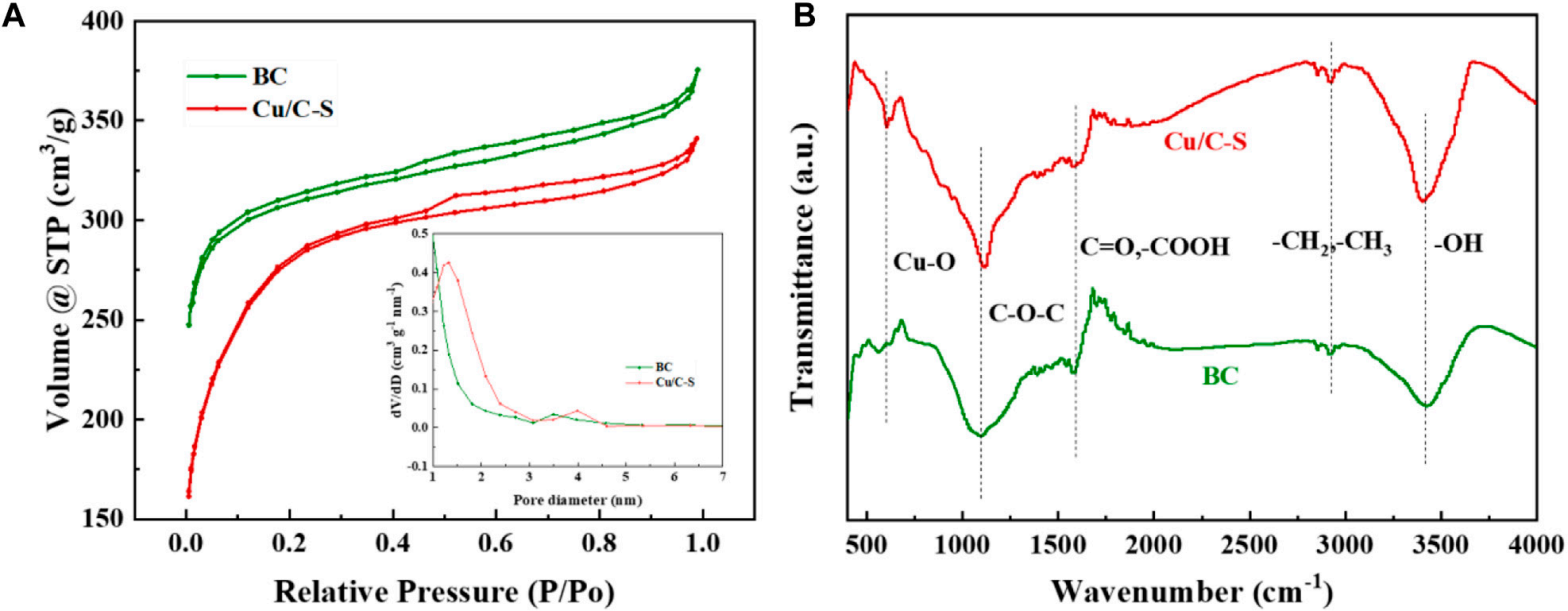
**(A)** Isothermal adsorption and desorption curves of BET; **(B)** FT-IR plots.

Figure 3A shows the nitrogen adsorption and desorption isotherms of pristine BC and Cu/C-S, both of which belongs to type IV in the IUPAC classification, with H1 hysteresis rings and typical mesoporous adsorption characteristics. According to Brunauer Emmett Teller (BET) theory and Barrett Joyner Halenda (BJH) adsorption model, specific surface area, pore volume and pore size were obtained (Agrafioti et al., 2013), and the results were shown in Supplementary Table S1. The specific surface areas of BC and Cu/C-S were 1202.9 m^2^/g and 1005.8 m^2^/g, respectively. The specific surface area and pore volume of the Cu/C-S decreased slightly and the pore size increased slightly after doping with Cu. In addition, as shown in Supplementary Figures S1, S2, the electrochemically active surface area (ECSA) of the material were determined by using the capacitance method, and the results showed that the ECSA of the Cu/C-S material was 3.4 times larger than that of the BC material, which may be caused by the large number of active sites provided by the surface-loaded elements of Cu/C-S. Figure 3B shows the Fourier Transform infrared spectroscopy (FT-IR) of pristine and Cu/C-S, respectively. The characteristic peak of Cu-O was detected at 600 cm^-1^ for the Cu/C-S material, indicating the oxide formation of Cu on the surface of CuSO_4_-modified activated carbon (He et al., 2020). The C-O-C stretching vibration peak near 900–1300 cm^-1^, the -OH peak appearing at 3449 cm^-1^ were enhanced for Cu/C-S materials, and the -CH_3_, -CH_2_ peak at 2,950–2,860 cm^-1^ and the C=O, -COOH peak at 1710 cm^-1^ are approximately unchanged (Fan et al., 2018; Zhu et al., 2019).

**FIGURE 4:**
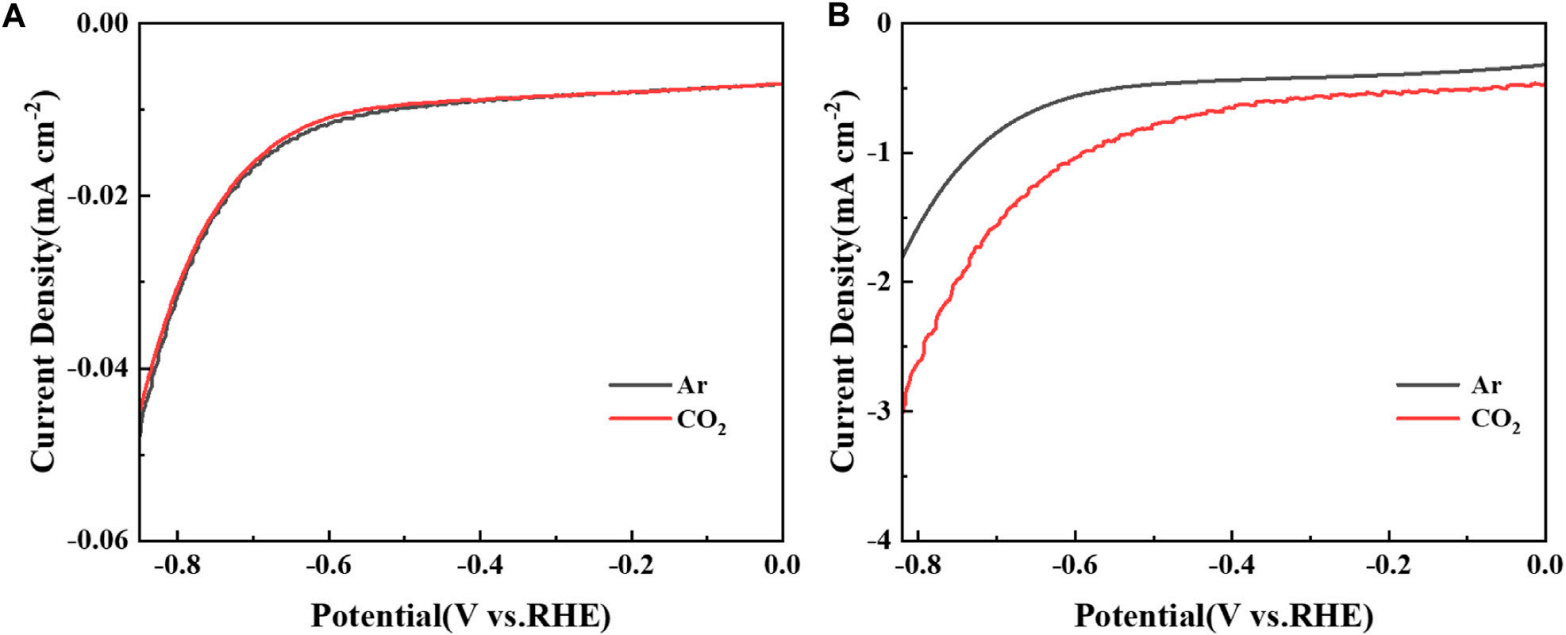
LSV of **(A)** BC and **(B)** Cu/C-S in 0.2 M Na_2_SO_4_ solution.

Figure 4 shows the linear sweep voltammetry (LSV) curves of the BC and Cu/C-S material electrodes in 0.2 M Na_2_SO_4_ solution at a scan rate of 10 mV/s. Without CuSO_4_ modification, the LSV curves of BC material in the atmosphere of argon and CO_2_ were shown in Figure 4A, which were basically identical, and the current density was very small (<0.05 mA/cm^2^). It could be concluded that tiny amount of HER dominated the system, and no significant CO_2_RR was observed. This may be due to the limited amount of copper in the directly harvested *Elsholtzia Harchowensis* from the mine, thus making it difficult to observe a significant electrocatalytic CO_2_RR reaction. The LSV curves of Cu/C-S materials under argon and CO_2_ atmospheres were shown in Figure 4B. The experimental results showed that the onset potential was −0.74 V (@1.0 mA/cm^2^) under argon atmosphere and −0.61 V (@1.0 mA/cm^2^) under CO_2_ atmosphere. In addition, under the same potential conditions, the current density in CO_2_ atmosphere was significantly higher than that in argon saturated system. This may be due to the fact that under the condition of CO_2_ purging in Na_2_SO_4_ solution, not only HER occurred, but also CO_2_RR happened at the same time, which contributed to additional current during the electrochemical process. Thus it could be concluded that Cu/C-S had a better electrochemical activity towards CO_2_RR.

**FIGURE 5:**
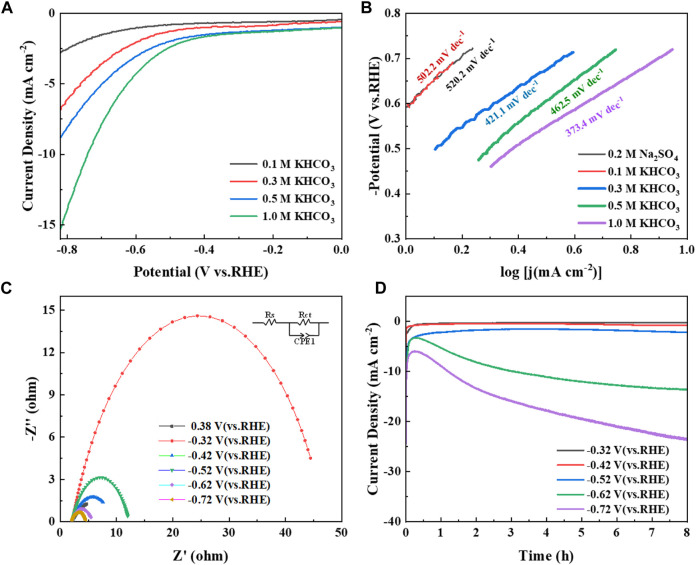
Electrochemical test plots of Cu/C-S materials. **(A)** LSV plots in different concentrations of KHCO_3_ solution; **(B)** slope of tafel curves in 0.2 M Na_2_SO_4_ as well as in different concentrations of KHCO_3_ solution; **(C)** impedance plots in 1 M KHCO_3_ solution; **(D)** i-t curves at different potentials.

Figure 5A shows the LSV curves of the Cu/C-S material electrode in different concentrations of KHCO_3_ solution at a scan rate of 10 mV/s. It can be seen from the figure that when the concentration of KHCO_3_ solution increased from 0.1 M to 1.0 M, the current density increased continuously and the onset potential of the reaction shifted significantly, which indicated a gradual increase in the electrocatalytic reaction activity. In addition, the tafel curves of the electrocatalytic process at different KHCO_3_ concentrations were shown in Figure 5B, which showed that the tafel slope in the KHCO_3_ solution was much smaller than that in the Na_2_SO_4_ solution, and the Tafel slope gradually decreased as the concentration of the KHCO_3_ solution increased. The tafel slope in 1.0 M KHCO_3_ solution was 373.4 mV‧dec^−1^, which indicated a faster growth of current density and better electrocatalytic performance, so the 1.0 M KHCO_3_ solution was subsequently chosen as the electrolyte solution. Figure 5C shows the impedance test results of Cu/C-S electrocatalytic reduction of CO_2_ under different potential conditions, and the results showed that the charge transfer resistance gradually decreased with the negative shift of potential. The material impedance was 7.9 Ω at the open circuit potential of 0.38 V (vs. RHE), 44.9 Ω at −0.32 V (vs. RHE) and 2.6 Ω at −0.72 V (vs. RHE) (see Supplementary Table S2 for detailed data), which indicated that the Cu/C-S material had good electrocatalytic CO_2_ reduction activity with low charge transfer resistance for CO_2_RR. Figure 5D showed the i-t curves at different potentials, which showed a trend of decreasing, then increasing and then stabilizing, with the initial rapid decay of current related to the end of double-layer charging. With the negative shift of potential, the current density gradually increased. The catalyst still has a good catalytic effect after 8 h of stable catalytic CO_2_ reduction, which indicated that the catalyst had certain stability in practical application.

**FIGURE 6:**
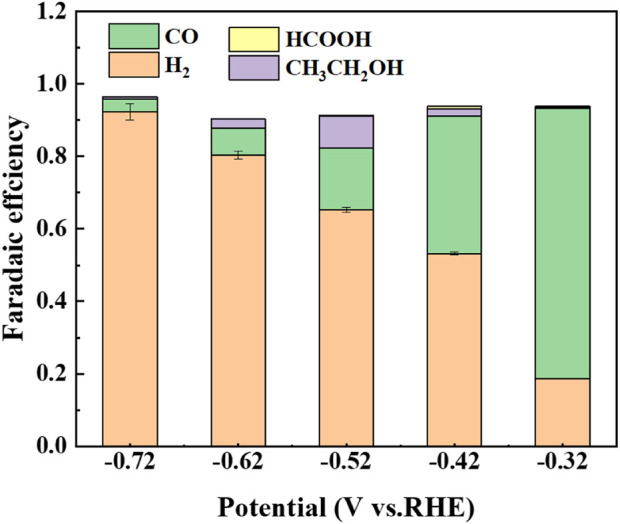
Product distribution of Cu/C-S materials for electrocatalytic carbon dioxide.

Figure 6 shows the distribution of final products by using Cu/C-S electrode at different potentials. The main gas phase products were H_2_ and CO, while the liquid phase products were formic acid and ethanol. CO_2_ was mainly converted to CO at −0.32 V (vs. RHE), with a high Faraday efficiency of 74.6% and a turnover frequency (TOF) of 107.2 h^-1^. Faraday efficiency of 8.7% for generating C_2_ products could be achieved at −0.52 V (vs. RHE). Pristine carbon materials were usually inert for electrochemical CO_2_RR and the introduction of S elements in the material may provide a large number of active sites and enhance the electrochemical activity of carbon materials (Liu et al., 2015; Asefa, 2016; Zhu et al., 2019). The Faraday efficiency of hydrogen generation was highest at a potential of −0.72 V (vs. RHE). Meanwhile, a trace amount of formate was generated after the reaction (see Supplementary Table S3 for detailed data). *COOH played an important role in the reaction process as a key intermediate in the CO_2_RR process (*CO_2_^−^ + H^+^→*COOH). In terms of CO conversion efficiency and TOF of the present material, the present catalyst was superior to most of the catalysts presented in Supplementary Table S4.

**FIGURE 7:**
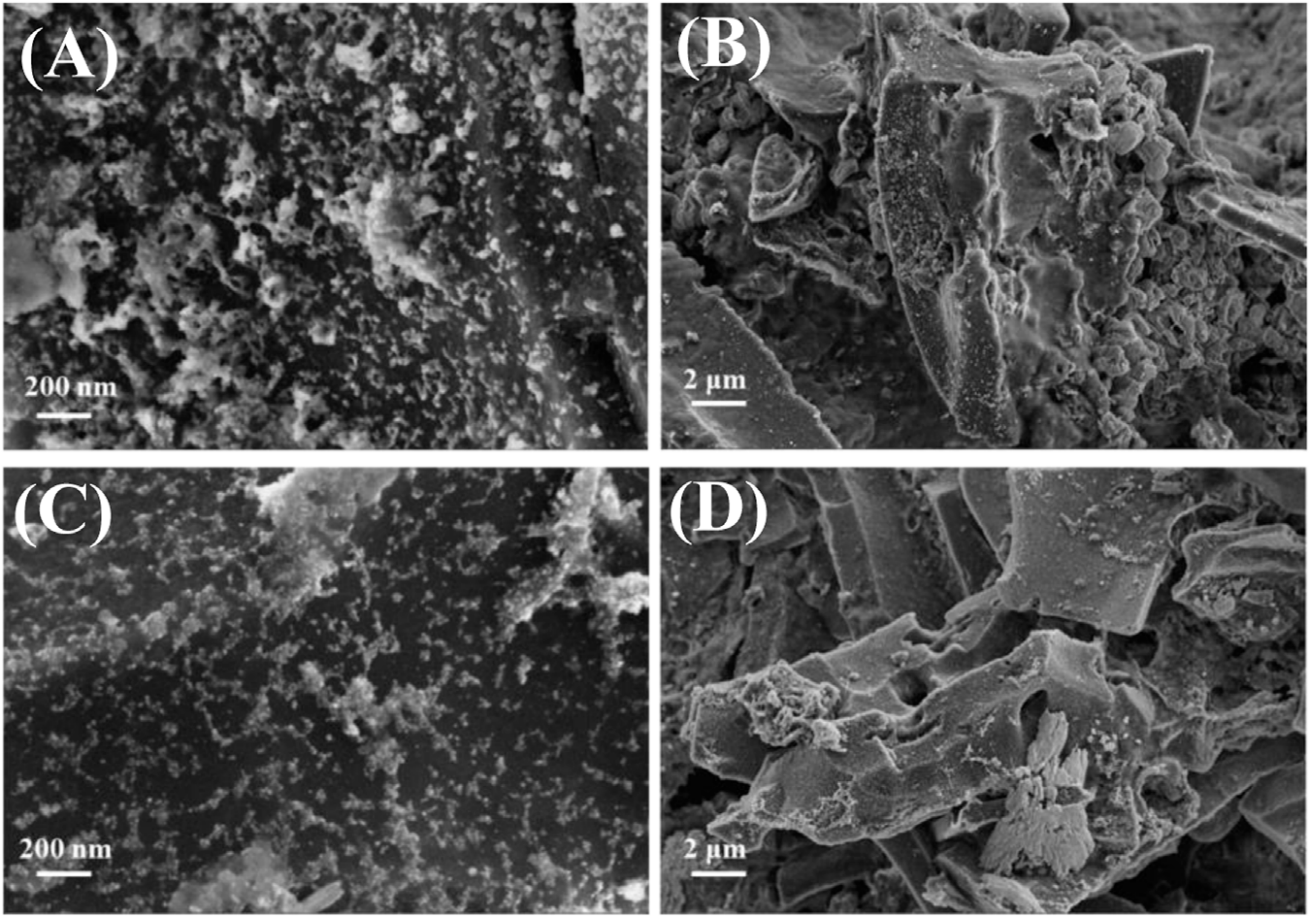
SEM images of Cu/C-S material before and after electrochemical testing. **(A, B)** are before the test; **(C, D)** are after the test.

**FIGURE 8:**
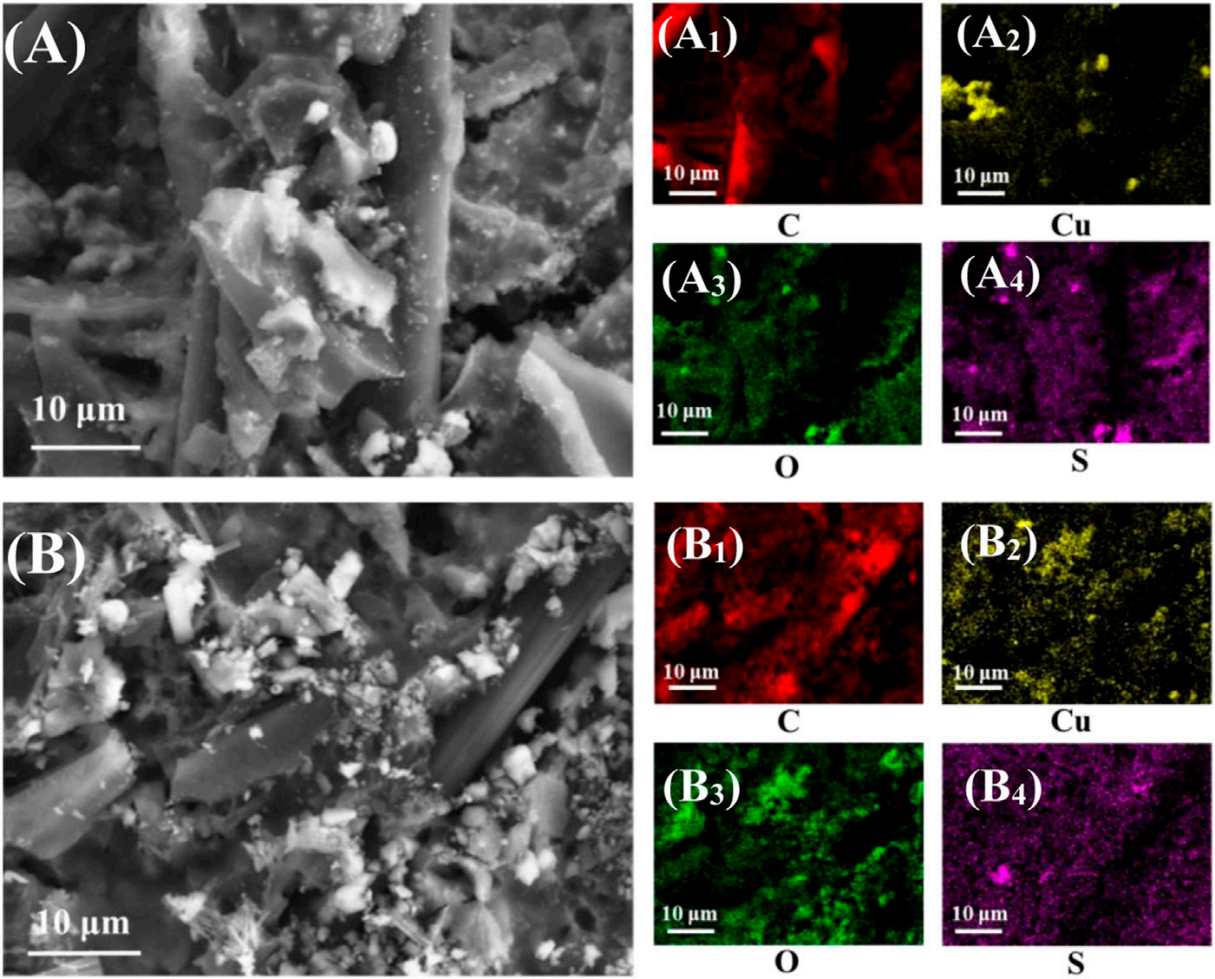
EDS-elements mapping of Cu/C-S material before and after electrochemical testing. **(A)** before the test, **(A**_**1**_ **–A**_**4**_ **)** are elemental spectra; **(B)** after the test, **(B**_**1**_ **–B**_**4**_ **)** are elemental spectra.

In order to continue to investigate the stability of the materials and the changes in the valence and surface functional groups during the reaction, the Cu/C-S materials before and after the electrochemical tests were characterized by SEM-EDS, XRD, XPS, and ICP-MS, respectively. The surface morphologies before and after the electrochemical tests were shown in Figures 7A–D, respectively. It can be seen from the figure that the catalyst loaded on the carbon cloth had a certain laminar structure with a relatively rough surface covered with more spherical particles, and the catalyst was uniformly distributed on the surface of the carbon cloth, which was consistent with the results of TEM, indicating that the black particles aggregated by TEM were compounds of copper. The catalyst morphology was almost unchanged before and after the test, with a slight reduction of particulate matter on the carbon cloth surface, probably due to the shedding of a small amount of material during the reaction. Plants were rich in elements such as C and O, while Cu and S were introduced in the synthesis process. Figures 8A,B showed the EDS-elements mapping scans before and after the test (see Supplementary Figure S4 for EDS layered images). The Cu/C-S material had a relatively uniform distribution of all four elements C, O, Cu, and S before and after the test, and the mass ratio of Cu element is around 7.0% (see Supplementary Figure S5 for the total spectrum of the elemental distribution map). There was a small amount of Cu element aggregation before the test, and the distribution of Cu was more uniform after the test, which better provided active sites CO_2_RR in the electrochemical process, could possibly be the reason for the gradual increase of current density in the i-t curve at more negative potentials during the electrochemical test.

**FIGURE 9:**
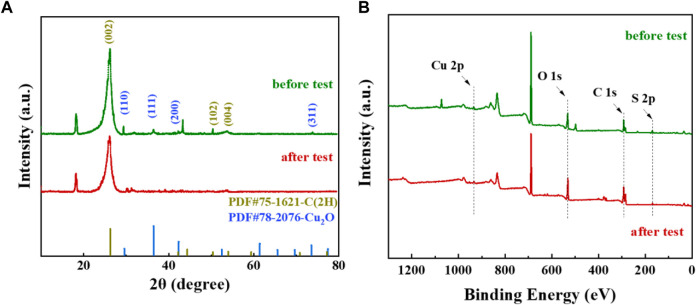
XRD and XPS plots of Cu/C-S materials before and after electrochemical testing. **(A)** XRD plots; **(B)** XPS plots.

**FIGURE 10:**
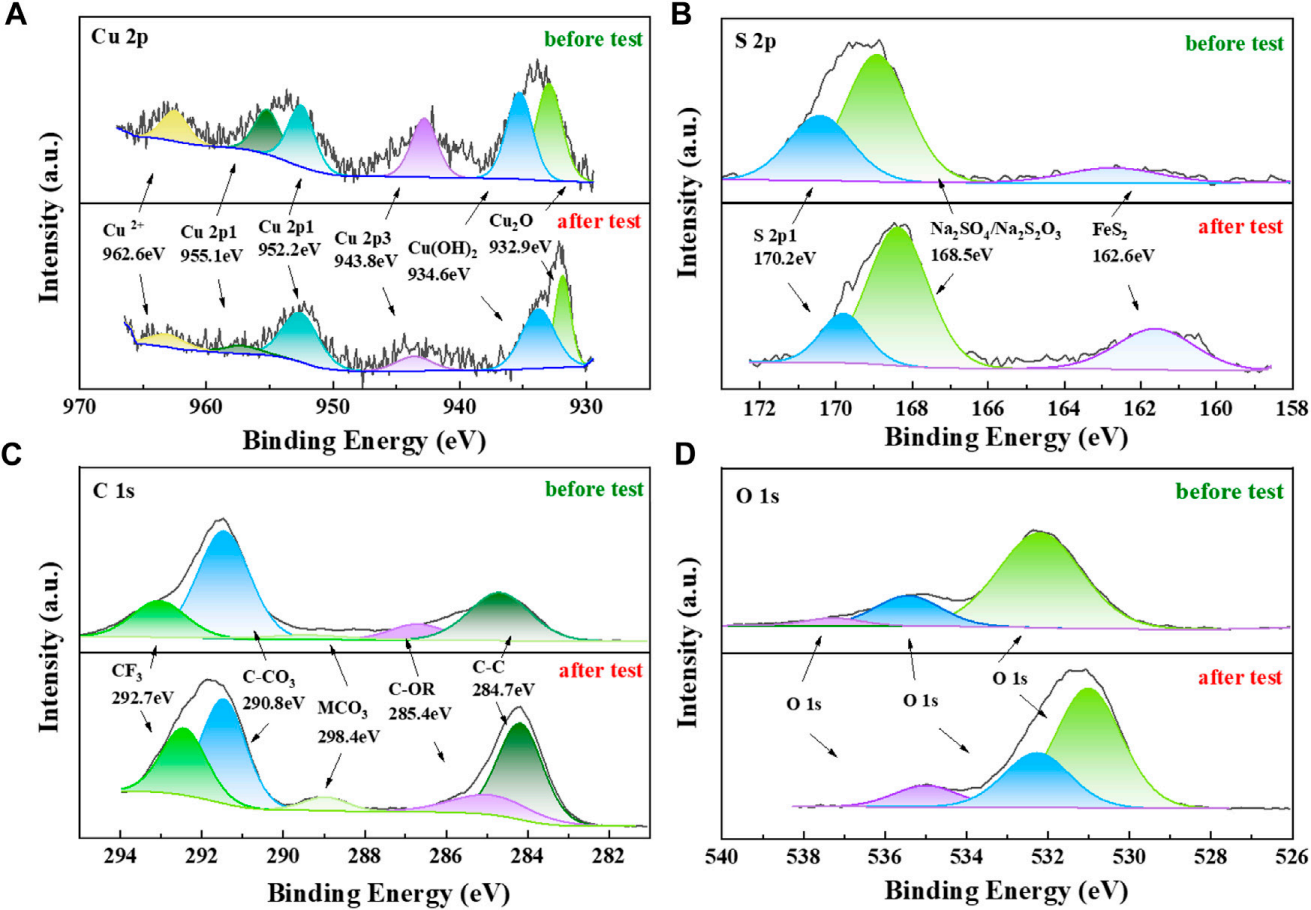
XPS fine spectra of Cu/C-S materials before and after electrochemical tests for **(A)** Cu 2p, **(B)** S 2p, **(C)** C 1s, and **(D)** O 1s.

In order to better understand the crystallographic characteristics of Cu elements on the material surface, the samples were analyzed using XRD, and the test results are shown in Figure 9A. The results showed that the Cu/C-S material had a graphitic carbon as well as Cu_2_O crystal structure, and the percentage of Cu_2_O crystals was relatively small. The diffraction peaks at 2θ of 26.2°, 50.3°, 54.0° corresponded to the (002), (102), and (004) crystal planes of graphitic carbon, and the diffraction peaks at 2θ of 29.6°, 36.4°, 42.3°, 73.6° correspond to the (110), (111), (200), and (311) crystal planes of Cu_2_O (Ren et al., 2015). Several major crystallographic peaks were basically unchanged before and after the reaction, indicating that the material structure remained relatively stable during the electrochemical process. The metallic contents of the as-prepared materials were measured by ICP-MS after microwave digestion. The results confirmed that the Cu/C-S material had Cu, Fe, Zn, Mn, Cr, Sb, Pb and other heavy metals, of which the metal Cu content was 37.9 mg/g as shown in Supplementary Table S5. As for the source of heavy metals such as Fe, Zn, Mn, Cr, Sb, and Pb, it may be due to the presence of these heavy metals in the soil, which were adsorbed and transferred to the plant tissue. Figure 9B shows the XPS full spectrum of the material, which validated the elemental composition as well as the chemical valence state of the sample. The results further demonstrated the presence of C, O, Cu, and S in the material. The fine spectra of C, Cu, S, and O elements (Figure 10) show that the peak position of Cu 2p was basically unchanged before and after the reaction, the element Cu exists mainly in the form of Cu^II^, Cu^I^, and the Cu_2_O peak at the binding energy of 923.9 eV echoed the FT-IR as well as XRD results, which confirms the existence of Cu_2_O. The production of Cu_2_O is first due to the impregnation method, where Cu^2+^ in the solution accumulates in plants through ion exchange or absorption by plant tissue cells. After preparation into electrode materials, the reduction at −0.82 V (vs. RHE) potential for 3600 s resulted in the partial reduction of Cu^2+^ to Cu_2_O. The peak percentage of Cu_2_O before and after the reaction is approximately unchanged, which indicates that Cu_2_O exists in a relatively stable form and can continuously electrocatalytically convert CO_2_ together with Cu^2+^ in the catalytic process (Wang et al., 2016; Liu et al., 2021). The fine spectrum of S 2p shows a peak of FeS_2_ at a binding energy of 162.6 eV, which was consistent with the XRD results. It shows that some S element combines with metallic elements to form metal sulfides, while non-metallic anions can provide vacancies for metal cations, which can change the electronic structure of metal cations, thus providing more active site for catalytic reactions (Guo et al., 2022). Due to the high content of element C in the material, many kinds of compounds were generated under high temperature, most of them exist in the form of C-C and C-O, and the peak proportion remains basically unchanged before and after the catalytic reaction. In summary, the XPS results corroborate the SEM results, and the structure and elemental valence of the material remain basically unchanged before and after the long catalytic reaction, with excellent catalyst stability performance.

## 4 Conclusion

In summary, the carbonaceous materials prepared from *Elsholtzia Harchowensis* had a large specific surface area and porous structure. The copper sulfate solution modified biochar had excellent electrocatalytic reduction of CO_2_ with instantaneous current density up to 15.3 mA/cm^2^ at −0.82 V (vs. RHE) electrode potential in 1.0 M KHCO_3_ solution. When the potential was −0.32 V (vs. RHE), the product CO Faraday efficiency could reach as high as 74.6%. At a potential of −0.52 V (vs. RHE), the C_2_^+^ product ethanol Faraday efficiency was 8.7%. The modified biochar prepared from *Elsholtzia Harchowensis* can be used as electrode material to realize solid waste utilization and convert carbon dioxide into useful products to achieve environmental protection as well as carbon reduction. This research work can provide new ideas for the utilization of heavy metal-enriched plants and explore the reuse of solid waste to achieve the dual objectives of environmental protection and economy.

## Data Availability

The original contributions presented in the study are included in the article/Supplementary Material, further inquiries can be directed to the corresponding author.
