# Supplementary material for: The synthesis of copper-modified biochar from Elsholtzia Harchowensis and its electrochemical activity towards the reduction of carbon dioxide
Source: Front Chem. 2023 Aug 30;11:1238424. doi: 10.3389/fchem.2023.1238424 (PMC10499400; doi:10.3389/fchem.2023.1238424)
Supplement: Supplementary file 1 [file DataSheet1.docx]

Supplementary Material

The synthesis of copper-modified biochar from *Elsholtzia Harchowensis* and its electrochemical activity towards the reduction of carbon dioxide

Shiqi Chen^1^, Wei Liu^1^, Ziwei Mei^1^, Haifu Li^1^, Wenyu Zhao^1^, Junkai Zhao^1^, HongTao^1,2^

*** Correspondence:** Corresponding Author: email@uni.edu

# Supplementary Electrochemical test

The catalyst was first activated by reduction in 0.2 M Na_2_SO_4_ solution at a potential of -0.82 V (vs. RHE) for 3600 s. All electrode potentials (vs. Ag/AgCl) in the experiments could be converted to standard electrode potentials (vs. RHE) by equation [1]. The cyclic voltammetry (CV) in 0.2 M Na_2_SO_4_ solution with various scan rates, and the final results could be got through equation [2] (Wei et al., 2019; Zhong et al., 2022).

$\boldsymbol{E(vs. RHE)= E(vs. Ag/AgCl)}\boldsymbol{+}\boldsymbol{0.209 V + 0.05916 \times pH}$ [1]

$\boldsymbol{ECSA=}\frac{\boldsymbol{C}_{\boldsymbol{dl}}}{\boldsymbol{C}_{\boldsymbol{s}}}\boldsymbol{=}\frac{\boldsymbol{I}_{\boldsymbol{c}}}{\boldsymbol{v\cdot}\boldsymbol{C}_{\boldsymbol{s}}}$ [2]

where *C_dl_* is the doubled-layer capacitance of the catalyst, *I_c_* is the average value of the charging current, and *v* is the scan rates. Linear sweep voltammetry (LSV) was performed at a scan rate of 10 mV/s within the range of 0 ~ -0.82 V(vs. RHE). Electrochemical impedance spectroscopy (EIS) was tested at a voltage of -0.32 ~ -0.72 V (vs. RHE), within frequency ranges of 10^5^ ~ 0.1 Hz, and an amplitude wave of 5 mV. Turnover frequency (TOF) is calculated by equation [3] (Hua et al., 2022):

$\boldsymbol{TOF=}\frac{{\boldsymbol{I}_{\boldsymbol{product}}}/\boldsymbol{nF}}{{\boldsymbol{\alpha\times}\boldsymbol{m}_{\boldsymbol{cat}}}/{\boldsymbol{M}_{\boldsymbol{metal}}}}\boldsymbol{\times3600}$ [3]

*I_product_* is the partial current density of certain product, n is the number of electrons for the conversion of CO_2_ to certain product, *F* is the Faraday constant (96485 C/mol), *m_cat_* is the mass of the catalyst, *α* and *M_metal_* are the mass ratio of active atoms in the catalyst and the atomic mass of the metal, respectively.

# Supplementary Figures and Tables

## Supplementary Figures

**
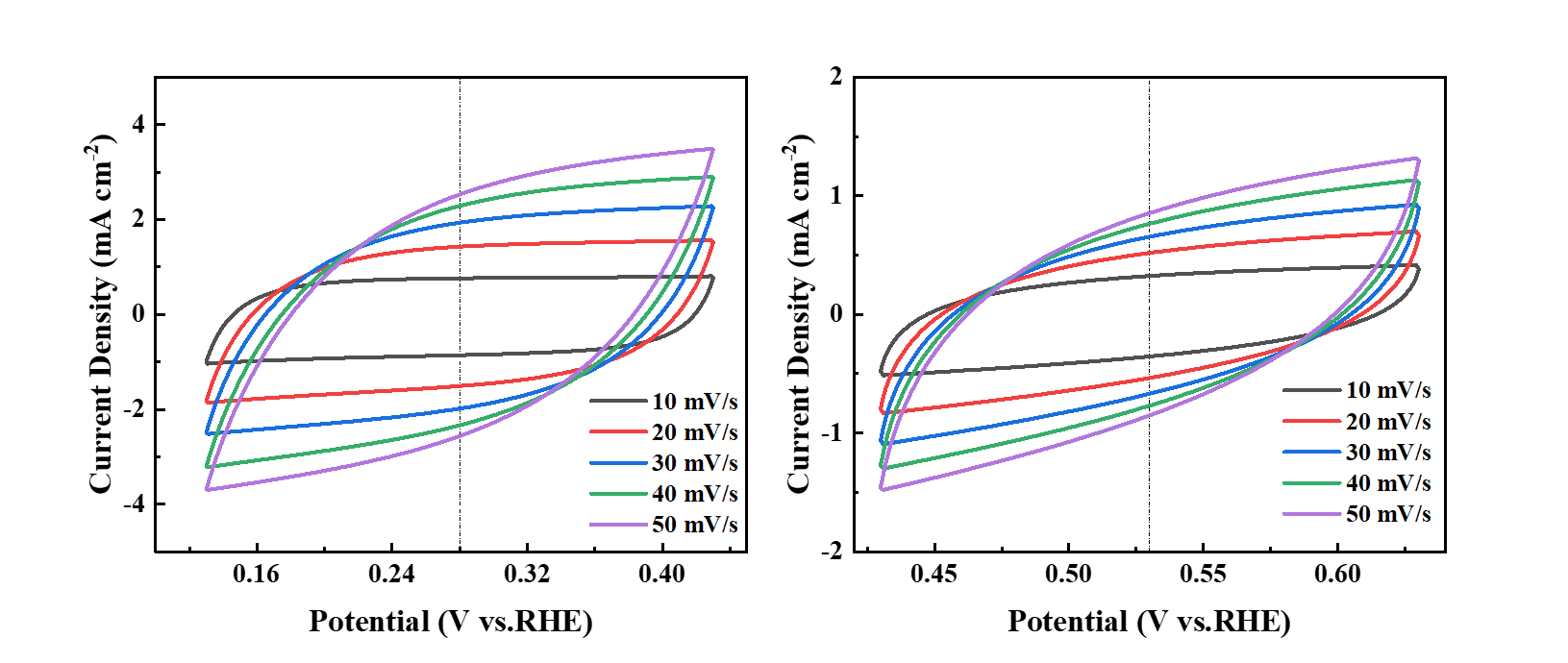
**

**b**

**a**

**Supplementary Figure 1.** CV curves of BC(a) and Cu/C-S(b) materials in 1 M KHCO_3_ solution at different sweep rates.

**a**

**
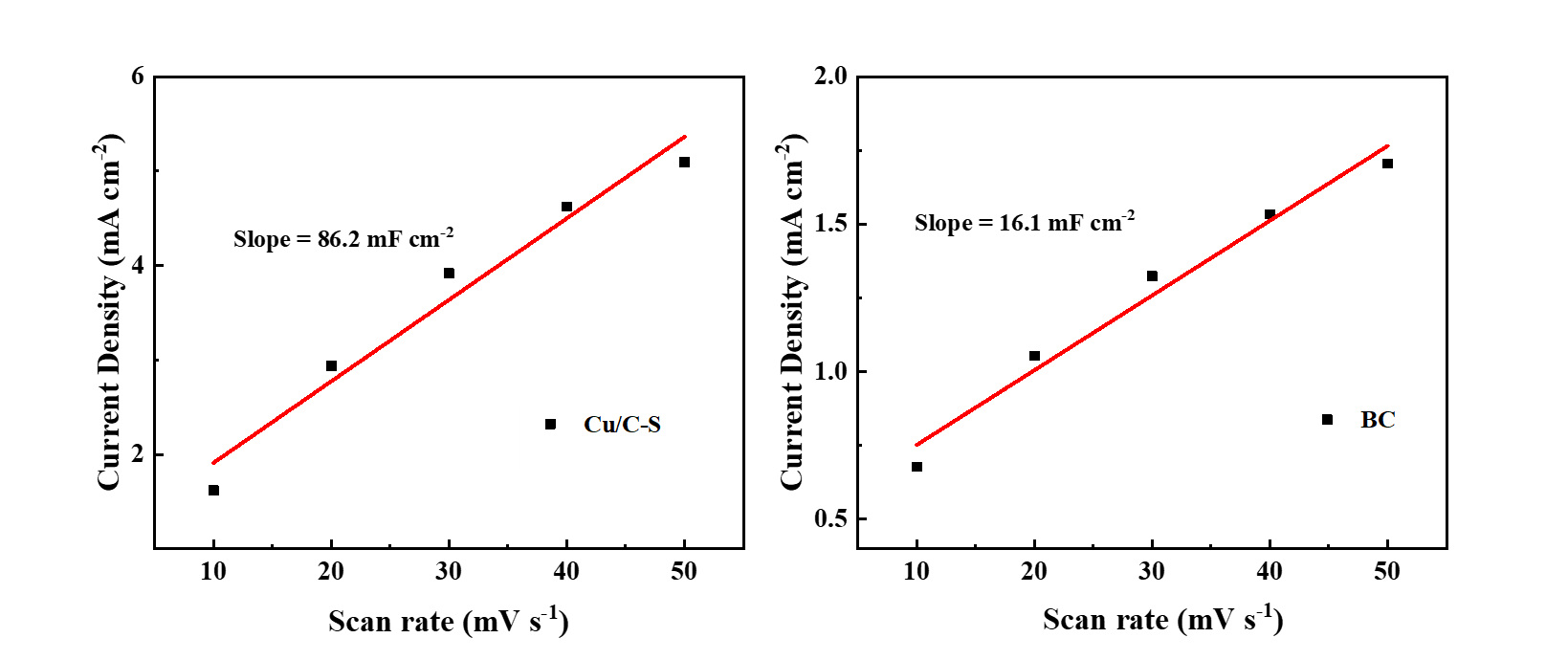
**

**b**

**Supplementary Figure 2.** Electrochemical activity area of BC(a) and Cu/C-S(b) materials.

**
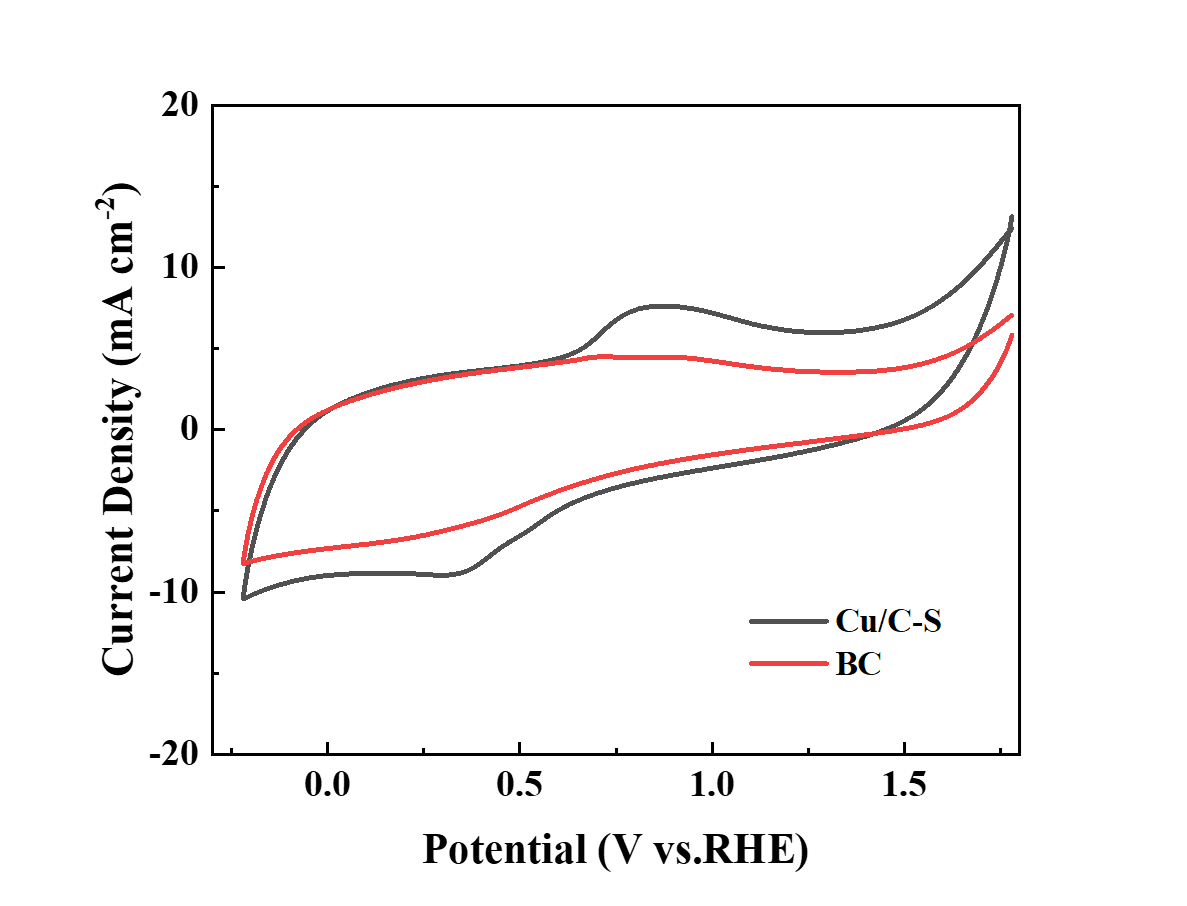
**

**Supplementary Figure 3.** CV curves of BC and Cu/C-S materials in 0.2 M Na_2_SO_4_ solution with a sweep rate of 50 mV/s.

**a**

**b**

**
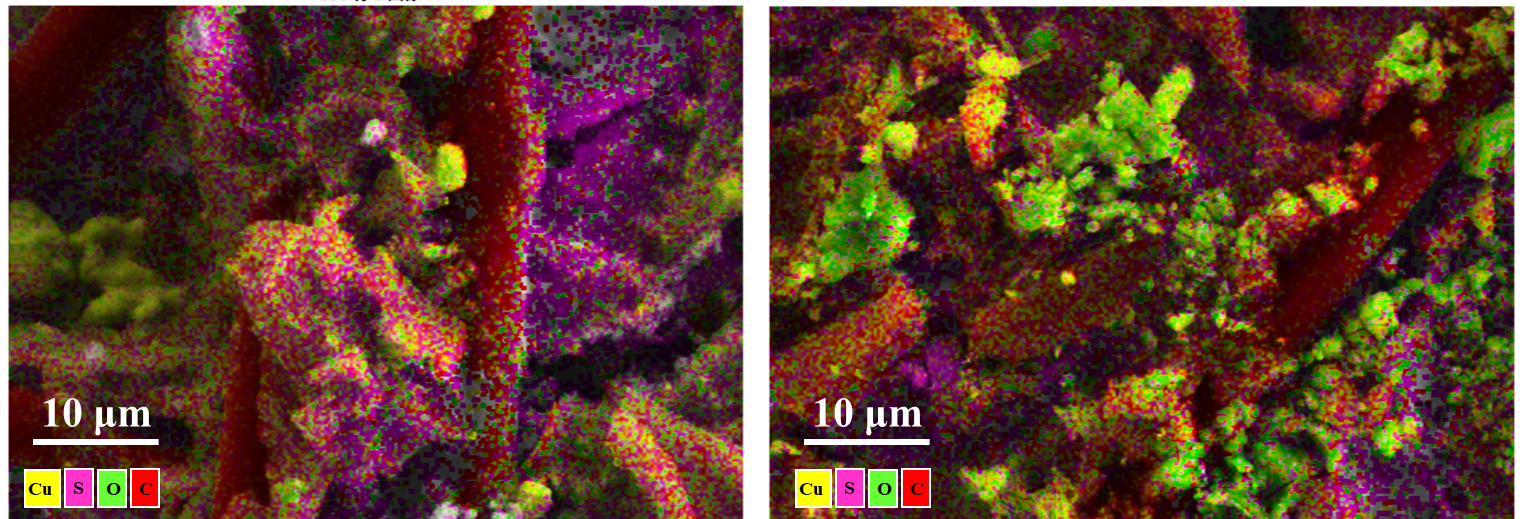
**

**Supplementary Figure 4.** EDS layered images of Cu/C-S material before and after electrochemical testing. a, before testing; b, after testing.

**a**

**b**

**
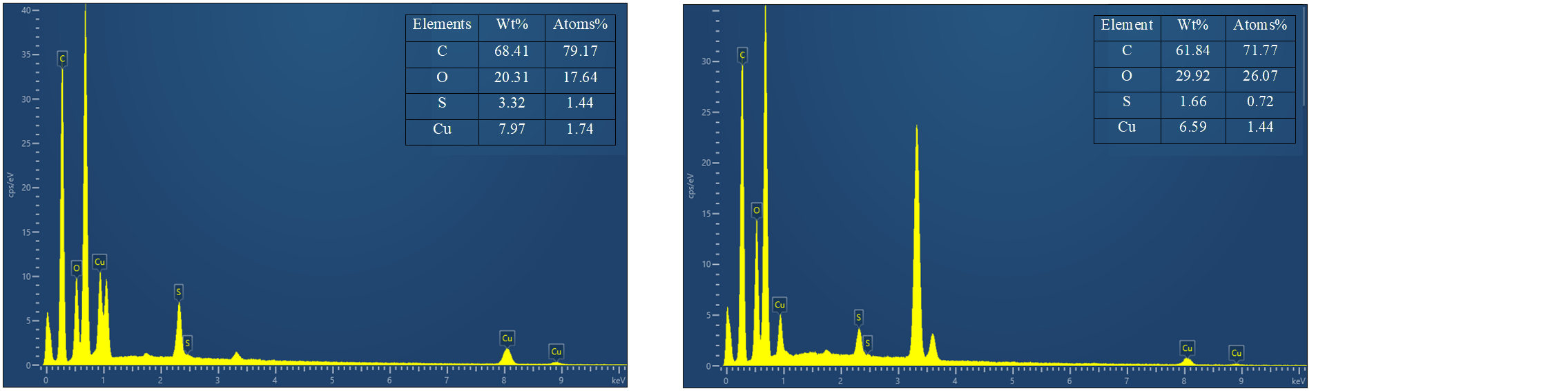
**

**Supplementary Figure 5.** Total spectra of elemental distribution maps of Cu/C-S materials before and after electrochemical tests. a, Before the test; b, After the test.

**
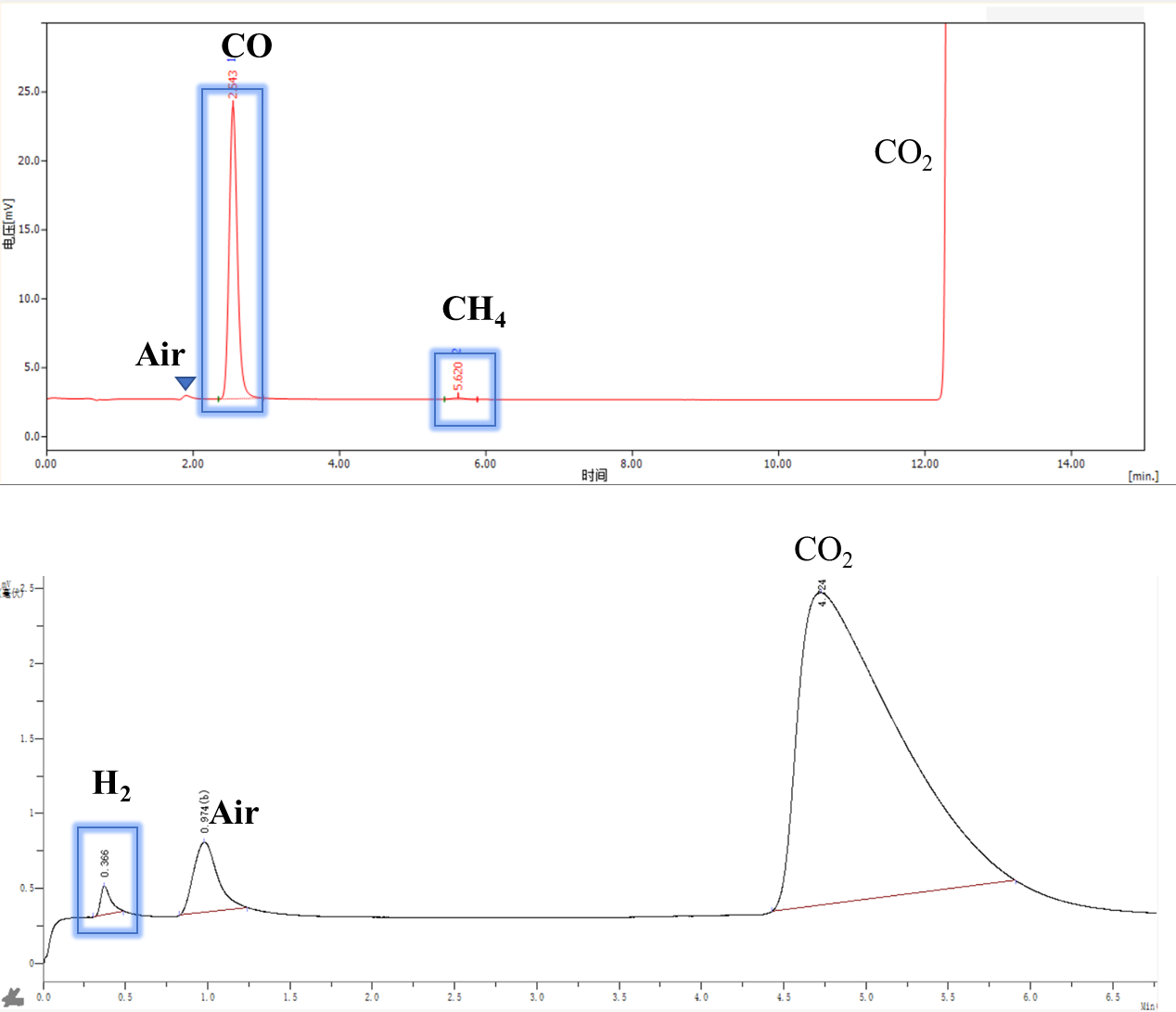
**

**a**

**b**

**Supplementary Figure 6.** Typical GC chromatogram of the gas phase product after electrolysis. a, FID detector; b, TCD detector.


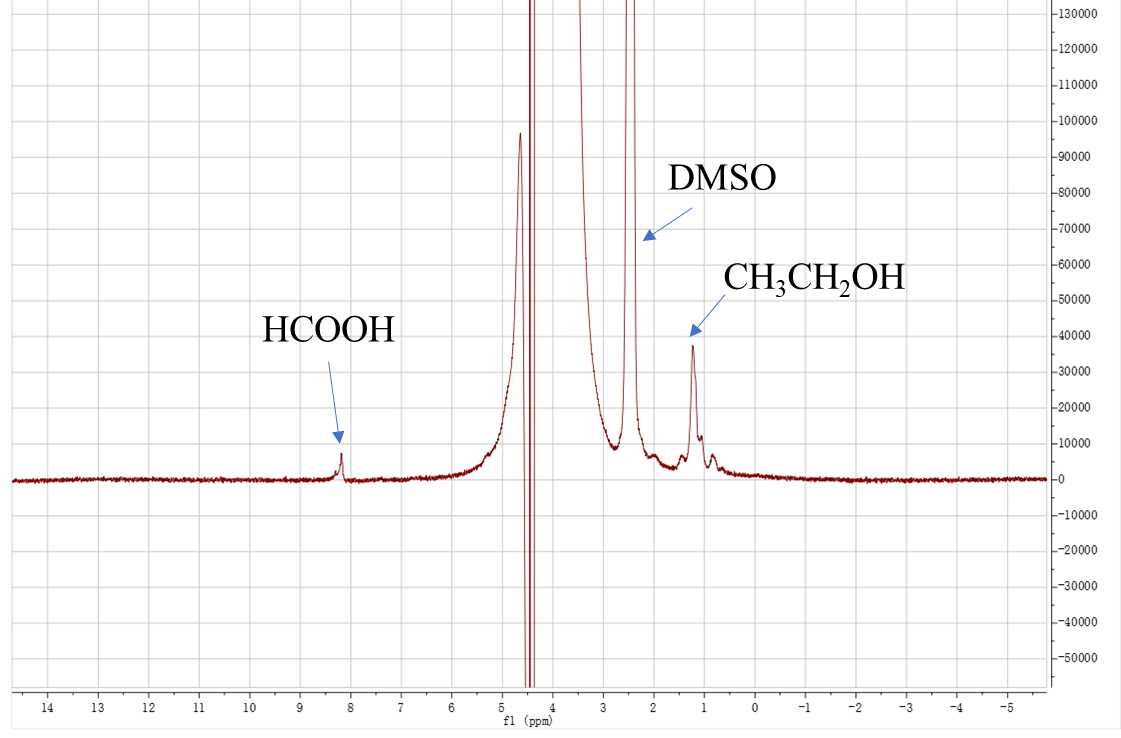


**Supplementary Figure 7.** Typical 1H NMR spectra of liquid phase products after electrolysis.

## Supplementary Tables

**Supplementary Table 1.** Specific surface area, pore volume, and pore diameter of BC and Cu/C-S materials.

| Materials | Surface Area  (m^2^/g) | Total pore volume  (cm^3^/g) | Average pore Diameter  (nm) |
| --- | --- | --- | --- |
| BC | 1202.9 | 0.58 | 1.93 |
| Cu/C-S | 1005.8 | 0.53 | 2.09 |

**Supplementary Table 2.** EIS fitting data for Cu/C-S material in 1.0 M KHCO_3_ solution.

| Potential  V(vs.RHE) | R_S_(Ω) | R_CT_(Ω) | CPE-T | CPE-P |
| --- | --- | --- | --- | --- |
| 0.38 | 2.06 | 7.86 | 0.050 | 0.43 |
| -0.32 | 2.17 | 44.88 | 0.0038 | 0.73 |
| -0.42 | 2.03 | 7.48 | 0.018 | 0.56 |
| -0.52 | 2.14 | 10.04 | 0.0042 | 0.71 |
| -0.62 | 2.02 | 3.69 | 0.013 | 0.58 |
| -0.72 | 2.03 | 2.56 | 0.0086 | 0.62 |

**Supplementary Table 3.** Product efficiency of Cu/C-S materials in 1.0 M KHCO_3_ solution at different potentials.

| Potential  V(vs.RHE) | H_2_  FE | CO  FE | CH_4_  FE | CH_3_CH_2_OH  FE | HCOOH  FE |
| --- | --- | --- | --- | --- | --- |
| -0.72 | 92.25% | 3.65% | 0.14% | 0.55% | 0.02% |
| -0.62 | 80.36% | 7.40% | 0.21% | 2.53% | 0.08% |
| -0.52 | 65.29% | 17.05% | 0.80% | 8.72% | 0.23% |
| -0.42 | 53.20% | 37.87% | 1.56% | 2.07% | 0.68% |
| -0.32 | 18.57% | 74.61% | 1.89% | 0.28% | 0.45% |

**Supplementary Table 4.** Comparison of CO_2_RR performance of Cu/C-S material and other materials.

| Catalyst | Potential V(vs.RHE) | FE  (%) | TOF  (h^-1^) | Electrolyte | Ref |
| --- | --- | --- | --- | --- | --- |
| Cu/C-S | -0.32 | 74.6(CO) | 116.4 | 1.0 M KHCO_3_ | This work |
| Cu/C-S | -0.52 | 8.7(C_2_H_5_OH) |  | 1.0 M KHCO_3_ | This work |
| Cu-Cu_2_O-1/Cu | -0.4 | 32(C_2_H_5_OH) | 15.6 | 0.5 M KHCO_3_ | (Zhu et al., 2019) |
| Cu_3_Ag_1_ | -0.95 | 63(C_2_H_5_OH) | / | 0.5 M KHCO_3_ | (Lv et al., 2020) |
| FeNC(ZIF-8) | -0.57 | 98.8(CO) | / | 1.0 M KHCO_3_ | (Hu et al., 2019) |
| Fe-N-C | -0.49 | 83(CO) | 410 | 0.1 M KHCO_3_ | (Pan et al., 2018) |
| Cu(core)/CuO(shell) | -1.78 (vs.Ag/AgCl) | 21(CO)  20(HCOOH) | / | 1.0 M KHCO_3_ | (Lan et al., 2014) |
| Cu_2_O | -0.99 | 39(C_2_H_5_OH) | / | 0.1 M KHCO_3_ | (Ren et al., 2015) |
| Ni−N-PC | -0.8 | 90(CO) | / | 0.5 M KHCO_3_ | (Gang et al., 2020) |
| Fe-N/P-C | -0.45 | 98(CO) | 508.8 | 0.5 M KHCO_3_ | (Li et al., 2022) |
| Ag_NP_/MnO_2_ | -0.90 | 68(CO) | / | 0.5 M KHCO_3_ | (Zhang et al., 2021) |
| Cu/In_2_O_3_ | -0.70 | 68(CO) | / | 0.5 M KHCO_3_ | (Xie et al., 2018) |

**Supplementary Table 5.** ICP-MS measurement data for BC and Cu/C-S materials

|  | Cu  (mg/g) | Fe  (mg/g) | Zn  (mg/g) | Mn  (mg/g) | Cr  (mg/g) | Sb  (mg/g) | Pb  (mg/g) |
| --- | --- | --- | --- | --- | --- | --- | --- |
| BC | 0.061 | 0.19 | 0.052 | 0.017 | 0.010 | 0.00030 | 0.0080 |
| Cu/C-S | 37.87 | 0.025 | 0.025 | 0.015 | 0.015 | 0.00026 | 0.0095 |

Gang, Y., Pan, F., Fei, Y., Du, Z., Hu, Y.H., and Li, Y. (2020). Highly efficient nickel, iron, and nitrogen codoped carbon catalysts derived from industrial waste petroleum coke for electrochemical CO2 reduction. *ACS Sustainable Chemistry Engineering* 8(23)**,** 8840-8847. doi: <https://doi.org/10.1021/acssuschemeng.0c03054>.

Hu, C., Bai, S., Gao, L., Liang, S., Yang, J., Cheng, S.-D., et al. (2019). Porosity-induced high selectivity for CO2 electroreduction to CO on Fe-doped ZIF-derived carbon catalysts. *ACS Catalysis* 9(12)**,** 11579-11588. doi: <https://doi.org/10.1021/acscatal.9b03175>.

Hua, Y., Zhang, B., Hao, W., and Gao, Z.J.C.R.P.S. (2022). Boosting CO desorption on dual active site electrocatalysts for CO2 reduction to produce tunable syngas. *Cell Reports Physical Science* 3(1)**,** 100703. doi: <https://doi.org/10.1016/j.xcrp.2021.100703>.

Lan, Y., Gai, C., Kenis, P.J., and Lu, J. (2014). Electrochemical reduction of carbon dioxide on Cu/CuO core/shell catalysts. *ChemElectroChem* 1(9)**,** 1577-1582. doi: <https://doi.org/10.1002/celc.201402182>.

Li, K., Zhang, S., Zhang, X., Liu, S., Jiang, H., Jiang, T., et al. (2022). Atomic tuning of single-atom Fe–N–C catalysts with phosphorus for robust electrochemical CO2 reduction. *Nano Letters* 22(4)**,** 1557-1565. doi: <https://doi.org/10.1021/acs.nanolett.1c04382>.

Lv, X., Shang, L., Zhou, S., Li, S., Wang, Y., Wang, Z., et al. (2020). Electron‐deficient Cu sites on Cu3Ag1 catalyst promoting CO2 electroreduction to alcohols. *Advanced Energy Materials* 10(37)**,** 2001987. doi: <https://doi.org/10.1002/aenm.202001987>.

Pan, F., Deng, W., Justiniano, C., and Li, Y. (2018). Identification of champion transition metals centers in metal and nitrogen-codoped carbon catalysts for CO2 reduction. *Applied Catalysis B: Environmental* 226**,** 463-472. doi: <https://doi.org/10.1016/j.apcatb.2018.01.001>.

Ren, D., Deng, Y., Handoko, A.D., Chen, C.S., Malkhandi, S., and Yeo, B.S. (2015). Selective electrochemical reduction of carbon dioxide to ethylene and ethanol on copper (I) oxide catalysts. *Acs Catalysis* 5(5)**,** 2814-2821. doi: <https://doi.org/10.1021/cs502128q>.

Wei, C., Sun, S., Mandler, D., Wang, X., Qiao, S.Z., and Xu, Z.J. (2019). Approaches for measuring the surface areas of metal oxide electrocatalysts for determining their intrinsic electrocatalytic activity. *Chemical Society Reviews* 48(9)**,** 2518-2534. doi: 10.1039/C8CS00848E.

Xie, H., Chen, S., Ma, F., Liang, J., Miao, Z., Wang, T., et al. (2018). Boosting tunable syngas formation via electrochemical CO2 reduction on Cu/In2O3 core/shell nanoparticles. *ACS Applied Materials Interfaces* 10(43)**,** 36996-37004. doi: <https://doi.org/10.1021/acsami.8b12747>.

Zhang, N., Zhang, X., Tao, L., Jiang, P., Ye, C., Lin, R., et al. (2021). Silver single‐atom catalyst for efficient electrochemical CO2 reduction synthesized from thermal transformation and surface reconstruction. *Angewandte Chemie International Edition* 60(11)**,** 6170-6176. doi: <https://doi.org/10.1002/anie.202014718>.

Zhong, X., Liang, S., Yang, T., Zeng, G., Zhong, Z., Deng, H., et al. (2022). Sn Dopants with Synergistic Oxygen Vacancies Boost CO2 Electroreduction on CuO Nanosheets to CO at Low Overpotential. *ACS nano* 16(11)**,** 19210-19219.

Zhu, Q., Sun, X., Yang, D., Ma, J., Kang, X., Zheng, L., et al. (2019). Carbon dioxide electroreduction to C2 products over copper-cuprous oxide derived from electrosynthesized copper complex. *Nature Communications* 10(1)**,** 3851. doi: <https://doi.org/10.1038/s41467-019-11599-7>.
